# A case report of medical laboratory confirmation of suspected scabies outbreak at Daara Madina Suwaneh, a quranic school located in Brufut Heights, The Gambia, 2025

**DOI:** 10.1186/s12879-026-12566-6

**Published:** 2026-01-16

**Authors:** Abdoulie M. Sanyang, Modou Colley, Lamin Sanneh, Mustapha Baldeh, Kebba Jadama, Abou Kebbeh, Alphonse Mendy, Saikou Maffuge Fatajo, Sheriffo Jagne, Amadou Woury Jallow, Momodou Nyassi, Bakary Sanneh

**Affiliations:** 1 National Public Health Laboratories, Department of Parasitology, Ministry of Health, Bertil Herding Highway, Kotu Layout (Same Complex as Central Medical Stores), Banjul, The Gambia; 2 Brufut Health Centre, Ministry of Health, Banjul, The Gambia; 3 Epidemiology and Disease Control Programme, Ministry of Health, Banjul, The Gambia; 4 Directorate of Health Services, Ministry of Health, Banjul, The Gambia; 5 Regional Health Directorate, Ministry of Health, Western Region 1, Banjul, The Gambia

**Keywords:** Scabies, *Sarcoptes scabiei*, Mite, Skin diseases, Simple saline mount, Skin scrapings, Scabies outbreak, The Gambia

## Abstract

**Background:**

Scabies is a high-burden neglected tropical disease, particularly in crowded, resource-limited settings, where children are disproportionately affected. Confirmation of scabies includes the use of clinical diagnosis supported by laboratory confirmation. This study was conducted to provide a laboratory confirmation for a clinically suspected scabies outbreak.

**Methods:**

Skin scrapings were collected from 13 clinically suspected cases and transported to the National Public Health Laboratories. Samples were processed using the simple saline mount technique. A case was considered positive when a mite, egg, or fecal pellet was identified with microscopy.

**Case presentation:**

A suspected scabies outbreak was reported from Daara Madina Suwaneh, a quranic boarding school located in Brufut Heights in the West Coast Region. A team from the Ministry of Health was deployed to confirm the scabies outbreak. A total of 13 male children were screened, with a mean age of 11.6 years (SD = 1.94) and an average symptom duration of 4.3 weeks (SD = 0.48). The hands and knees were the most frequently affected sites, observed in 38.5% of cases. Laboratory analysis found mite eggs in only one child, and no mites were detected in any of the samples, resulting in a positivity rate of 7.7%. All the cases were treated with 10% Benzyl Benzoate lotion, as it is the recommended medication for the treatment of children by the World Health Organization, and they fully recovered. This report highlights the important role of a simple saline mount, a low-cost routine parasitological technique, in supporting clinical suspicion of a scabies outbreak.

**Conclusions:**

This laboratory confirmation has provided supportive evidence for clinical diagnosis of scabies at a quranic school in The Gambia. The low laboratory positivity likely reflects challenges associated with the use of the normal saline method for sample processing, instead of potassium hydroxide, the most reliable method. These findings highlight the importance of timely sampling and improved diagnostic capacity for disease outbreak confirmation in resource-limited settings.

## Introduction

Scabies is a neglected tropical disease (NTD) [1, 2] caused by the human itch mite *Sarcoptes scabiei* var. *hominis*, an ectoparasite that burrows into the skin leading to intense itching and rash due to hypersensitivity reactions triggered by the mite’s eggs, feces and secretions [3]. The lesions vary by age group and body site, disproportionately affecting infants, children, adolescents, and adults living in crowded and resource-poor settings [4]. *Sarcoptes scabiei* has various subspecies that are host-specific, and this study is specifically concerned with the variety that infects humans.

Globally, an estimated 158 million people are infected with scabies annually [2], with a prevalence ranging from 0.2 to 71% [1]. Furthermore, of the 202.6 million global prevalent scabies cases reported, children and young people bear the highest burden [5]. One study restricted to children revealed a prevalence of > 10–20% [6]. A study reported prevalence ranging from 4.7 to 33.7% in sub-Saharan Africa [7]. Prevalence among schoolchildren in Africa vary from country to country and setting to setting. For instances, studies conducted in a range of settings reported a prevalence of approximately 1.6% [8] in Senegal, ~ 10.8% in Ethiopia [9], ~ 11% attack rate in Ghana [10] and ~ 21.8% [11] in Nigeria. Further studies reported prevalence ranging from 71% in Ghana [12], 17.8% in Cameroon [13], 2.9% in Malawi [14], to 4.4% in Egypt [15]. Moreover, some studies have also reported a prevalence ranging from 11 to 33.5% [11, 16, 17] and 2.5% [18], respectively.

High occurrence rates are commonly observed in warm, tropical regions, particularly where poverty and overcrowded living conditions are prevalent. Therefore, preventive measures, including maintaining personal hygiene, avoiding contact with infected individuals, and treating clothing and bedding are crucial for controlling the spread of scabies.

Transmission of scabies occurs primarily through prolonged skin-to-skin contact typically lasting 15 to 20 min with an infected person, and less commonly through indirect contact with contaminated materials such as bedding, clothing, and furniture. Mites locate their hosts using odour and temperature gradients [19]. The incubation period following a first infection is usually 4–6 weeks, after which symptoms appear. These include intense nocturnal itching, a pimple-like rash, burrowing tracks, and secondary bacterial infections resulting from scratching. Commonly affected areas include the webs of the fingers, wrists, elbows, waist, buttocks, and genital regions.

Clinical diagnosis of scabies is not reliable and often leads to delayed intervention. This is because the disease manifests differently from one patient to another and symptoms mimic many common skin conditions. This is compounded by the limited sensitivity of simple confirmatory tests such as the simple saline method, which is often not readily available. These factors lead to frequent misdiagnosis, wrong treatment outcomes and delayed control interventions [20].

Confirmatory diagnosis for scabies includes but is not limited to dermoscopy, adhesive tape test and skin scraping techniques. Skin scraping is a widely practiced, low-cost diagnostic method commonly employed to confirm cases of scabies. The procedure entails carefully scraping material from affected skin sites, especially active burrows or papules, using a sterile scalpel blade. The diagnosis is confirmed by microscopic examination of scabies mites, eggs or faeces. Among these techniques, dermoscopy had the highest diagnostic sensitivity (97.5%) compared with 75% for skin scraping and 62.5% for the adhesive tape test. Dermoscopy also had higher specificity (91.7%) than skin scraping (83.3%) or the adhesive tape test (70.8%) [21].

The World Health Organization recommends the use of Benzyl benzoate as a first-line topical scabicide in resource-limited settings due to its efficacy, affordability and relative ease of use compared with Sulphur ointment. 10% Benzyl benzoate lotion is recommended for the treatment of children and 20–25% for adults. Therefore, 10% Benzyl benzoate was used in treating the children [22, 23].

In the present study, normal saline was used to prepare wet mounts because potassium hydroxide (KOH) and other keratolytic reagents were not available during the immediate field processing. While saline has been used in prior clinical reports [24], it is known to be less effective at clearing keratin than KOH and may therefore reduce the sensitivity for detecting mites and eggs.

Scabies is a notifiable disease under the Integrated Disease Surveillance and Response (IDSR) system due to its high transmissibility, potential for outbreaks, and substantial health and socio-economic impact. In The Gambia, despite its significance, available epidemiological data on scabies are limited, with only one recent notable study conducted among under five years restricted to peri-urban area with limited generalizability [25]. Availability and access to reliable epidemiological data are crucial for designing effective community and school-based public health intervention strategies, in order to have a better understanding of this disease prevalence [7, 15]. Given the limited laboratory capacity for scabies confirmation and the potential for a full-blown outbreak due to overcrowding in the quranic boarding school, a rapid response team was deployed from the Ministry of Health to provide epidemiological assessment. This study reports on the laboratory confirmation of the clinically suspected scabies outbreak at the quranic school.

## Methods

### Settings

Daara Madina Suwaneh is a quranic school located in Brufut Heights, a primarily residential community near the Atlantic coast and close to Brusubi and Brufut. The community is known for its peaceful environment and several guesthouses. The initial reports of the outbreak came from public media/news sources. This was followed by a visit to the quranic school by a rapid response team on the 30th of May 2025 between the hours of 11:00 and 13:00. The team comprised of a public health officer from Brufut Health Centre, two laboratory personnel and a driver from the National Public Health Laboratories (NPHL), Ministry of Health.

### Observation by the rapid response team

**Fig. 1:**
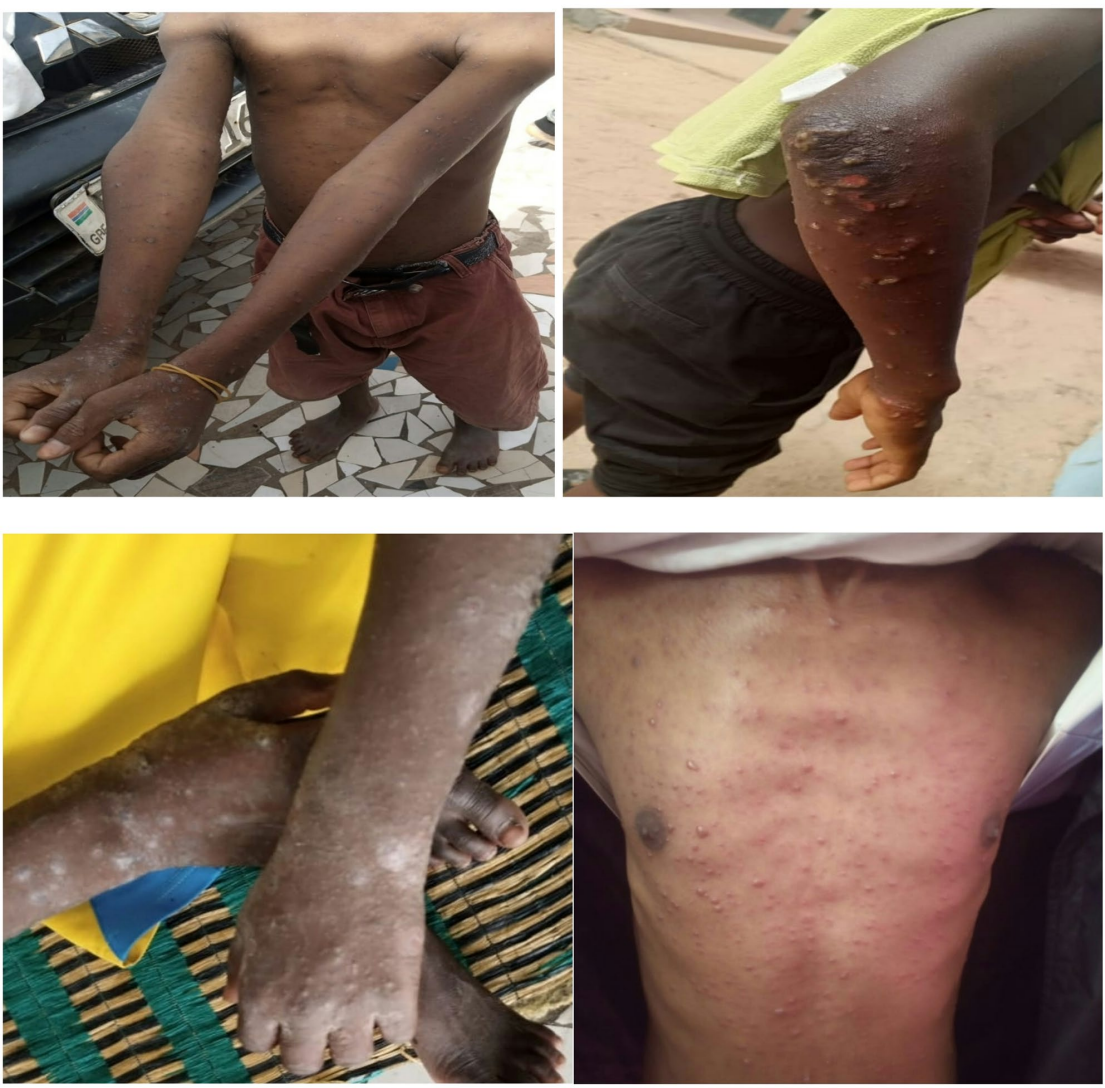
Scabies outbreak signs observed among students at Daara Suwaneh Quranic School, Brufut Heights, The Gambia

The team noted that most of the affected children are showing signs of recovery from scabies skin infection. Observations included fading rashes and lesions, healing burrows, peeling or flaking skin, and the absence of new lesions (Fig. 1).

### Inclusion criteria

This included any child that [1] resided in *Daara Madina Suwaneh* during the outbreak period [2], presented with clinical signs suggestive of scabies, such as intense nocturnal itching, papular rash, or visible burrows in typical sites (interdigital spaces, wrists, elbows or knees lesion), and [3] consent was obtained for from the head of the quranic school.

### Exclusion criteria

This included any child that [1] refused examination or sampling [2], had no visible skin lesions suitable for scraping, or had severe secondary bacterial infections that would interfere with safe sampling, and [3] who was not present at the time of the investigation.

### Sample size

The investigation was conducted as a rapid field response. The 13 samples represented all suspected cases who were present, consented, and had suitable lesions for scraping at the time of the visit.

### Detailed sampling procedure

Each participant was examined for the presence of active lesions by the investigation team. Scrapings were obtained from typical scabies sites such as wrists, elbows and knees. Using a sterile disposable scalpel blade, a small area (~ 5 mm²) at the edge of an active burrow or papule was gently scraped until superficial epidermal material was obtained. For each child, 2–3 scrapings were collected from interdigital spaces, wrists, elbows or knees lesion sites and pooled into a 15 mL sterile centrifuge tube. All samples were labelled with unique codes and transported to the Parasitology Reference Laboratory (PRL) in a sample transportation container within two hours of collection.

### Clinical history

Before the initiation of the sample collection, the investigation team obtained a brief history for each participant. Information included age, duration of symptoms, previous treatment for scabies or other skin conditions, and the body sites affected. Several children reported using Benzyl Benzoate lotion prior to the laboratory visit, which may have contributed to the healing appearance of lesions and the low detection rate in microscopy.

### Clinical evaluation

All suspected cases were clinically assessed once during the field visit, when the samples were collected. Clinicians and the desk public health officer from Brufut Health Centre jointly conducted treatment follow-up within two weeks, confirming that all children showed clinical recovery. No serial laboratory follow-up was performed as the investigation’s primary goal was initial outbreak confirmation.

### Data analysis

Data were entered into Microsoft Excel and analysed using descriptive statistical methods with the Statistical Package for the Social Sciences (SPSS), version 27. Results were expressed as means and standard deviations for age and duration of symptoms and as frequencies and percentages for categorical variables (lesion sites, positivity rates). No inferential statistical tests were applied due to the small sample size and descriptive nature of the investigation.

### Ethical considerations

The investigation formed part of routine public health outbreak response and was conducted under the authority of the Directorate of Health Services. Consent for sampling was obtained from the head of the institution and assent from participants as appropriate. Data were anonymised; no individual identifiers are reported.

### Laboratory investigation

#### Simple saline Mount

The samples collected were all processed and analysed after two hours of their collection at the Parasitology Reference Laboratory, NPHL, by three laboratory scientists. To maximize the chances of detecting mites or eggs, 2–3 scrapings were collected per participant from active lesions, particularly interdigital spaces, wrists and knees. The collected material was thoroughly mixed in saline, and three wet mounts were examined per sample under low power (10X) and high power (40X) objectives of a compound microscope CX21 (Olympus, Tokyo, Japan) and results were recorded in a reporting sheet. Each mount was screened independently by three experienced laboratory scientists. Microscopists recorded a slide as positive only when an egg, mite or fecal pellet was detected.

### Result

**Fig. 2:**
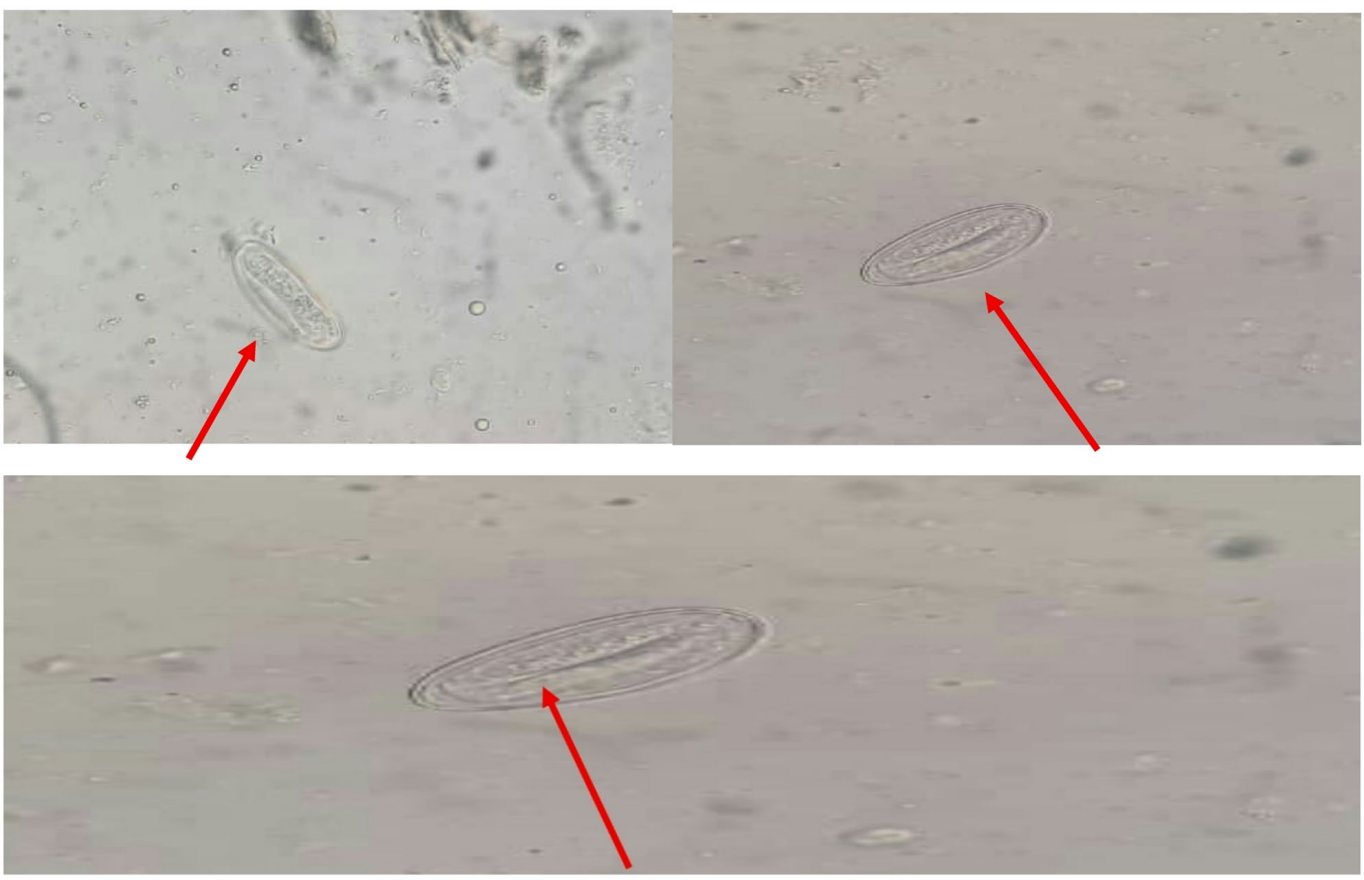
A 400x total magnification of a typical scabies egg from a simple saline mount observed in the sample of one of the children. The egg appears oval or elliptical, with a thin outer shell and visible internal granules under the microscope

Only one out of the thirteen samples tested positive for scabies egg, the remaining twelve children who tested negative microscopically were diagnosed negative based on characteristic lesion appearance, nocturnal itching, and distribution pattern consistent with scabies, supported by clinical assessment conducted by the outbreak investigation team using a standard case definition. The mean age of the tested individuals was 11.6 years (SD = 1.94) and the average duration of scabies symptoms was 4.3 weeks (SD = 0.48). Only male students were screened because the affected institution, Daara, is a predominantly a male boarding school where they all live in a crowded housing. This focus on male students was therefore based on the epidemiological evidence that the outbreak was confined to the student population. The most frequently affected areas were the hands and knees, observed in 5 children (38.5%). Eggs were detected in only one child (7.7%) (Fig. 2), and no mites were identified in any of the children (Table 1). The positivity rate was 7.6% (1/13).

**Table 1:** Demographics, clinical characteristics and laboratory confirmation results for the sampled children (*N* = 13)

Children	Age (years)	Gender	Location of scabies	Duration of scabies	Egg seen	Mite seen	Treatment
1	7	M	Hand, knee	4 weeks	No	No	Benzyl Benzoate lotion
2	13	M	Knee, Buttocks	5 weeks	No	No	Benzyl Benzoate lotion
3	13	M	Foot, hand	4 weeks	No	No	Benzyl Benzoate lotion
4	10	M	groin, hands	4 weeks	No	No	Benzyl Benzoate lotion
5	11	M	Hand, knee	4 weeks	No	No	Benzyl Benzoate lotion
6	10	M	Hand, knee	5 weeks	Yes	No	Benzyl Benzoate lotion
7	11	M	Groin, hand, knee	4 weeks	No	No	Benzyl Benzoate lotion
8	12	M	Groin, hand, Buttocks knee	5 weeks	No	No	Benzyl Benzoate lotion
9	11	M	Groin, hand, knee	4 weeks	No	No	Benzyl Benzoate lotion
10	14	M	Buttocks, hand, knee	4 weeks	No	No	Benzyl Benzoate lotion
11	13	M	Hand, knee	4 weeks	No	No	Benzyl Benzoate lotion
12	15	M	Hand, knee	5 weeks	No	No	Benzyl Benzoate lotion
13	12	M	Groin, hand, knee	4 weeks	No	No	Benzyl Benzoate lotion

## Discussion

This study provides a laboratory confirmation for a suspected scabies outbreak at a quranic boarding school in The Gambia. The study found a 7.7% prevalence of scabies among the 13 male students tested. This prevalence is lower than those reported by some studies, which recorded higher rates (> 10–20% [6], ~ 10.8% [9], ~ 21.8% [11], 17.8% [13], and 11- 33.5% [11, 16, 17]). The lower prevalence observed in the present study may be attributed to the reduced sensitivity of the microscopic examination method used for the skin scraping samples. In contrast, the 7.7% prevalence reported in this study is slightly higher than those reported by studies in Senegal (1.6%) [8], Malawi (2.9%) [14], Egypt (4.4%) [15] and Ethiopia (2.5%) [18].

Microscopic examination of skin scrapings remains the gold standard for the diagnosis of scabies. However, the method is relatively less sensitive and often unavailable in many settings, which can lead to misdiagnosis and delayed treatment [24], consequently facilitating the rapid spread of the disease. The preferred technique for detecting *Sarcoptes scabiei* is the use of potassium hydroxide (KOH), particularly in resource-limited environments.

A total of 13 male participants, aged 7–15 years (mean age 11.6 years), were examined during the outbreak investigation. The average duration of symptoms was about 4.3 weeks, with most children reporting symptoms lasting between four and five weeks. The hands and knees were the most commonly affected areas, observed in 38.5% (5/13**)** of the children, followed by the groin and buttocks as observed in many children. All children were treated with 10% benzyl benzoate lotion and showed clinical improvement during the follow-up period. The study only screened male students because the affected institution, Daara Madina Suwaneh, is predominantly a male boarding school, where children live in crowded housing. The teaching and non-teaching staff of the boarding school were also assessed during the investigation, but none showed signs or symptoms of scabies infection at the time of screening.

This study has several limitations. First, the simple saline mount technique was used as potassium hydroxide (KOH) was not available during field sample processing. As saline lacks keratolytic properties, we acknowledge this as a major methodological limitation, and it likely accounts, in part, for the low positivity rate observed in this study. Although Kandi (2017) previously employed the saline mount and demonstrated its usefulness [24] as an alternative to KOH, the method is however less effective at clearing keratinized material, thereby lowering the sensitivity for detecting mites and eggs, and potentially contributing to the low detection rate observed in this study. Second, cases might have been underreported due to the inability to use additional more sensitive techniques such as dermoscopy. Third, the study only examined the 13 samples out of the 57 clinically suspected cases. There is a likelihood that the study could have detected more cases had all the 57 suspected case samples were collected and examined. Fourth, there was no dermatologist in the investigation team that conducted the clinical assessment of the suspected cases. Fifth, all the schoolchildren in the study were male, which made it impossible to have information on the distribution of the disease across the genders. Future studies should adopt a more collaborative approach, include a dermatologist in the investigation team and utilize more sensitive diagnostic methods to ensure all suspected cases are effectively assessed clinically, samples collected and tested using robust methods.

## Conclusion

Our investigation revealed laboratory confirmation of scabies among the samples collected to support clinical and epidemiological evidence of an outbreak of the disease at a quranic boarding school in The Gambia. The low laboratory positivity likely reflects challenges associated with field conditions and the use of normal saline during sample preparation instead of potassium hydroxide (KOH), which is the standard practice for the microscopic detection of *Sarcoptes scabies*. The findings of the study highlight the need for timely sampling, use of the most reliable traditional parasitological methods and improved diagnostic capacity for outbreak confirmation in resource-limited settings, in order to increase the detection of mites, their eggs, and fecal pellets (scybala). Early detection and response to an outbreak are significant steps in curbing the spread of the disease. Therefore, future outbreak responses should prioritise the early engagement of key stakeholders, including health authorities, education officials, existing local governance structures and community leaders to strengthen and support intervention efforts.

## Data Availability

The original data for this report is available from the corresponding author.

## Abbreviations

IDSR: Integrated Disease Surveillance and Response
NTD: Neglected Tropical Disease
WHO: World Health Organization
PLOS: Public Library of Science
CLSI: Clinical and Laboratory Standards Institute
MRCG: Medical Research Council Unit The Gambia
SOPs: Standard Operating Procedures
MDA: Mass Drug Administration
GID: Gambia Immigration Department
EDC: Epidemiology and Disease Control
NPHL: National Public Health Laboratories
SD: Standard Deviation
ELIZA: Enzyme-linked immunosorbent assay
qPCR: Real-time quantitative polymerase chain
